# Optimal deployment of limited vaccine supplies to control mpox outbreaks

**DOI:** 10.1038/s41541-025-01289-5

**Published:** 2025-11-20

**Authors:** Matthew T. Berry, C. Raina MacIntyre, Deborah Cromer, Adam Hacker, Miles P. Davenport, David S. Khoury

**Affiliations:** 1 Infection Analytics Program, Kirby Institute, UNSW Sydney, Kensington, NSW 2052 Australia; 2 Biosecurity Program, Kirby Institute, UNSW Sydney, Kensington, NSW 2052 Australia; 3 College of Health Solutions, Arizona State University, Tempe, USA; 4 Coalition for Epidemic Preparedness Innovations, Post box 1030 Hoff, 0218 Oslo, Norway

**Keywords:** Viral infection, Predictive markers, Antibodies, Public health

## Abstract

Mpox outbreaks in central and East Africa have been declared a public health emergency of international concern by the World Health Organization. Fortunately, real-world effectiveness studies of the MVA-BN vaccine indicate that it has an effectiveness of 74% after one dose, and 82% after two doses against mpox. However, given the very limited availability of vaccines, there remain questions around the optimal deployment of limited MVA-BN doses. In this study, we consider whether more mpox cases might be averted by following the traditional two-dose vaccine regimen (4 week dosing interval), or by giving a single dose of MVA-BN to as many individuals as possible. We find that the optimal strategy depends on (i) the degree to which a subpopulation might be at higher risk of mpox, or severe mpox, and (ii) how long ago the first dose was administered to the most at-risk subpopulation.

## Introduction

There are concurrent outbreaks of mpox linked to clades I and II monkeypox virus (MPXV) globally^1^. Of great concern is the current clade I outbreak in central and East Africa with high case rates, increasing transmission and case-fatality ratio reported at ~2% (African continent average), and higher in children^2,3^. Fortunately, there is considerable evidence that vaccinia-based vaccines are effective at preventing mpox and reducing the severity of illness^4–6^. The evidence of vaccine effectiveness (VE) is derived from real-world effectiveness studies of the third generation MVA-BN vaccine in western populations during the global epidemic of mpox linked to clade IIb virus ongoing since 2022^7–10^. A number of older studies in the Democratic Republic of Congo (DRC, formally Zaire) have also shown that vaccination with first generation vaccinia-based vaccines is protective against mpox^11–13^. However, third generation vaccines are preferable to early vaccinia vaccines because of the superior safety and utility, particularly in immunosuppressed individuals^14^. Despite the urgent need to vaccinate at-risk populations, particularly in the DRC and neighbouring countries, vaccine availability is limited^15^. Africa CDC estimate 10 million doses are required to control the epidemic^16^. Japan has committed 3 million doses of LC16, with 50,000 having reached DRC or neighbouring countries. As of February 2025, just over 540,000 doses of MVA-BN (Jynneos) had arrived in Africa^17^. LC16 is a single dose regimen, while MVA-BN is currently recommended as a two-dose regimen (given 4 weeks apart)^18^. The DRC has a population of >110 million. Therefore, how to optimally deploy a limited supply of vaccines to avert the greatest number of clinical cases, particularly severe cases, is a critical question at present.

Similar questions have been considered in regard to COVID-19 vaccine deployment^19–22^, with a number of studies noting the dependence of optimal deployment on the relative effectiveness of one and two dose regimens^19,20^. Recently, multiple meta-analyses of real-world effectiveness studies of MVA-BN have analysed the effectiveness of one-dose and two-dose MVA-BN against clade IIb mpox^4–6^. These have shown a relatively incremental improvement in vaccine effectiveness after two doses compared with a single dose. For example, the World Health Organization (WHO) states that one dose and two doses of MVA-BN provide 76% and 82% effectiveness against mpox, respectively^23^. In our own meta-analysis we estimated very similar vaccine effectiveness of 74% after one dose and 82% after two doses. Based on this comparison, we commented that in the case of limited vaccine supply, it would be optimal to deploy a single dose of vaccine to as many individuals as possible rather than administer two doses to half as many individuals. However, this analysis did not include modelling of the waning of vaccine effectiveness with time after one dose vaccination (i.e. the comparison was based on reported vaccine effectiveness from clinical studies, and did not model the predicted decay of vaccine effectiveness over time). Further, the analysis only considered the situation of a population at homogeneous risk of infection, but did not consider the implications of high-risk groups for both infection and severe outcomes, whereas higher risk of infection and severe outcomes in younger age groups have been observed in the clade I outbreak in the DRC^2,24^.

Here we consider the question of the optimal deployment of a limited number of MVA-BN doses given the likely waning of vaccine effectiveness, and in a population where there are subgroups at different risk of infection and severe outcomes. In particular, we are interested in the decision, at a particular time point, of whether to use a limited supply of MVA-BN vaccines to deliver a second dose to those who have already received a first dose of MVA-BN or to deploy a first dose of the limited vaccine supply to naïve individuals. This question does not consider the reason individuals may have received a first dose in the past (e.g. either as post-exposure prophylaxis or as primary prevention), or the protection the first vaccine doses have provided up to the time our decision must be made. But rather, we only ask, from the time of our current decision on deployment, what is the relative advantage of deploying a limited supply of these vaccines as second doses to those already partially vaccinated or as first doses to those who have not yet received any vaccine doses?

It is important to note that, in this study, we rely on data and modelling of MVA-BN effectiveness derived from the clade IIb global outbreaks, but throughout our analysis we discuss these results assuming they are translatable to the contemporaneous clade I outbreaks in Africa. Although it is generally thought that these vaccines will also be effective against clade I MPXV (since immunity to orthopoxviruses is often cross-protective^14^), confirmatory studies showing that these vaccines are effective against clade I mpox are currently lacking, and are urgently needed. Further, in this analysis we do not explicitly model mpox transmission or consider the impact of different vaccination strategies on transmission or the epidemic trajectory, since there is limited data to inform these outcomes. Instead, we consider only the situation where vaccine supply is so limited that deployment decisions will not have a significant impact on overall transmission or force of infection in the community, and focus our analysis primarily on the goal of reducing risk of infection and severe outcomes in particular cohorts.

## Results

### Comparing one-dose versus two-dose MVA-BN vaccination strategies over 2 years

**Fig. 1:**
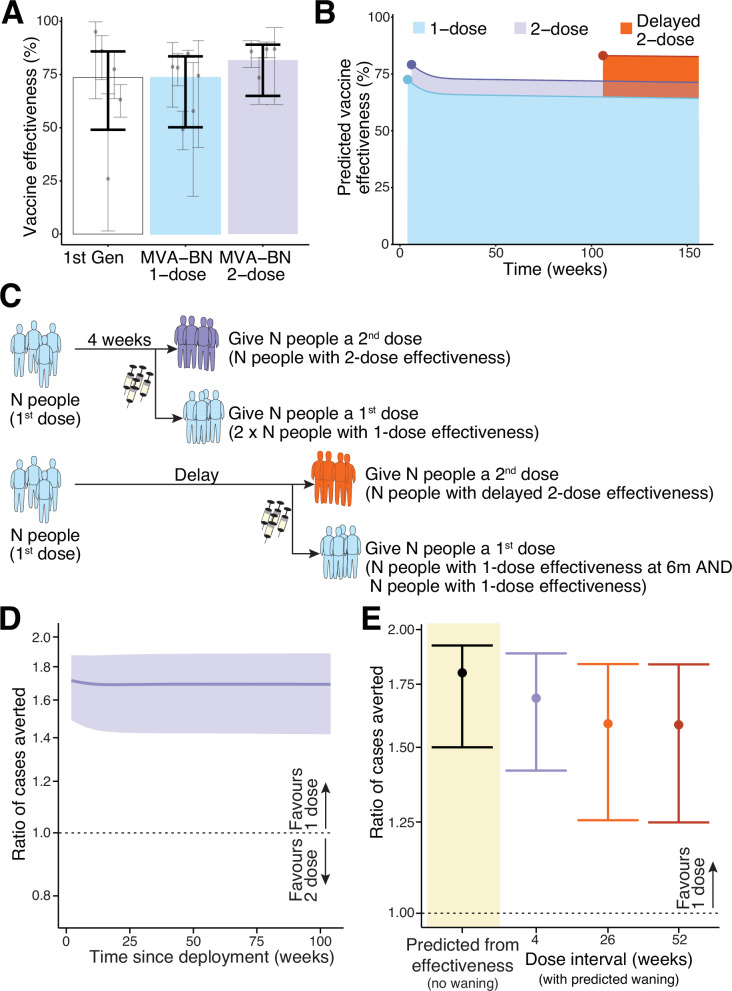
Estimating the protection from using MVA-BN in a one versus two-dose schedule. **A** The previously reported vaccine effectiveness of first-generation vaccinia immunisation, and one and two dose MVA-BN vaccination (against clade IIb virus) from a systematic review and meta-analysis of the available data^4^. **B** The predicted vaccine effectiveness over time for one dose, two doses spaced 4 weeks apart, and delayed two dose (administered at 2 years). These predictions are from reference^4^, where a model-based meta-analysis was performed that linked reports of vaccinia-binding antibody titres after MVA-BN vaccination and real-world effectiveness studies, and vaccine effectiveness over time was extrapolated by assuming these binding titres predict vaccine effectiveness (which is not yet confirmed). **C** Schematic of potential vaccine allocation options assuming additional vaccine is available 4 weeks after primary vaccination (top) or at some later timepoint (bottom). In each case we compare the outcome of either giving a second dose to those already vaccinated or allocating the additional vaccine as first doses to a naïve population. **D** The ratio of cases averted by administering a first dose to naïve individuals compared to a second dose (when deployment is 4 weeks after the first doses), measured over the first 2 years after vaccination. The ratio does not vary much over the time interval being considered. **E** The ratio of cases averted by giving a one dose regimen rather than a two-dose regimen, estimated using the vaccine effectiveness observed in a meta-analysis of real world effectiveness studies^4^ (left, black) or predicted from a model-based meta-analysis using predictions of waning and different dose spacing^4^. Note that the analysis here assumes a low and constant force of infection. Also, in the case of giving a second dose at 26 and 52 weeks we make the assumption that peak antibody binding titres and decay in titres will resemble that seen after giving a second dose at 2 years (the only delayed interval for which immunogenicity data was available^4^). This assumption inflates the predicted benefit of the two-dose regimen. Shaded regions (**D**) and error bars (**A**, **E**) indicate 95% credible intervals.

In a previous study we estimated that distributing limited doses of MVA-BN as a single dose regimen would avert around 1.8-fold more cases of mpox than deploying the same number of doses as a two-dose regimen^4^. This calculation was based only on the comparative reported effectiveness of the one and two dose regimens (Fig. 1A). However, vaccinia-binding antibody titres wane over time, and if antibody responses are predictive of protection, vaccine effectiveness against mpox may wane in parallel^4^ (Fig. 1B). Therefore, in this case, it is important to model the possible effect of this decay on the comparative benefits of a one- vs. two-dose regimen. We consider a scenario where one dose of vaccine has been deployed to some groups of individuals either as primary prevention or post-exposure prophylaxis, and at some later timepoint, public health authorities will have a choice to either administer a second dose to those same individuals already primed, or to administer a first dose of vaccine to naïve individuals (Fig. 1C). Without modelling the effects of waning immunity, using only the estimates of vaccine effectiveness from our previous meta-regression of real world effectiveness studies^4^, a one-dose vaccination schedule is predicted to avert 1.80-fold (CI: 1.50-1.92), as we reported previously. In order to account for the impact of waning vaccine effectiveness, we first consider a scenario of low and relatively constant transmission levels, as has been observed over much of the last decade^25^ (note that we also consider the more general case of a varying force of infection below). We then consider different vaccination strategies, accounting for the impact of vaccination or boosting at different times and the decay of vaccinia-binding antibody titres and protection after vaccination. The ratio of the cases averted with each strategy can be calculated as the ratio of the average vaccine effectiveness over a given time interval, beginning when the vaccines are deployed under one or the other strategy. This analysis suggests that the benefit of administering a first dose to as many people as possible is relatively constant over the first 2 years after vaccination (Fig. 1D). In the subsequent analysis we focus on a time interval of 2 years after deployment, by which time vaccine manufacturing and supply are likely to have increased.

The analysis above considers the scenario where sufficient vaccine is available to deliver a second dose 4 weeks after the first. However, if vaccine doses become available later after the first dose, does this change the relative benefit of a one-dose vs. a two-dose strategy? Studies have shown that delaying the second dose until 2 years after the first dose leads to a higher peak, and slower decay, of antibody titres post-vaccination^26^. Thus, in addition to declining protection from the initial one-dose vaccination over time, the relative benefit of administering a delayed second dose is expected to increase over time. To model this scenario, we take the same function of waning one-dose immunity as above (Fig. 1B). Of note, we only have data on the impact of increasing the spacing of two doses from 4 weeks to 2 years (Fig. 1B), and do not have detailed data on the effects of different dose-spacing intervals on antibody titres. However, we make the assumption that delaying the second dose to 26 weeks or more induces a similar peak immune response to that achieved with a second dose at 2 years (Fig. 1B), which is an assumption that is expected to overestimate the protection from the two-dose regimen when the second dose is administered at 26 or 52 weeks. After accounting for waning immunity and higher predicted protection with a delayed second dose, administering a first dose to as many individuals as possible is still predicted to maximise the cases averted from mpox compared to a two-dose strategy (Fig. 1E). This is observed whether additional vaccine becomes available, 4, 26 or 52 weeks after priming and despite our use of a conservative approach to estimating the effect of a delayed second dose (that will favour a two-dose regimen). We estimate there is still a 1.58-fold (CI: 1.25-1.84) advantage of administering first doses to naïve individuals compared to a strategy of administering a second dose to those already vaccinated individuals at 52 weeks.

### Accounting for a higher and variable force of infection

**Fig. 2:**
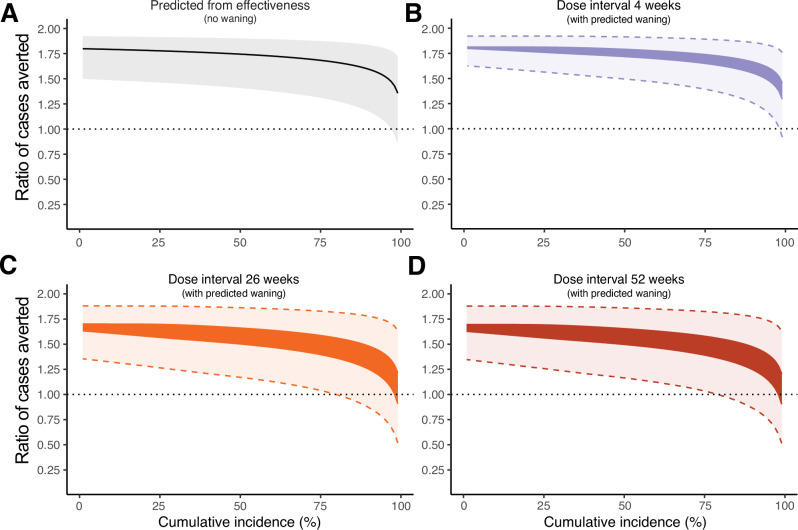
Ratio of cases averted with the one vs two-dose schedule and varying cumulative incidence. **A** Assuming a time-varying force of infection and non-decaying vaccine effectiveness, we predict the ratio of cases averted between the one-dose and two dose-strategies, assuming different cumulative incidences (% of people in the population infected in 2 years if unvaccinated). Solid line indicates the ratio of cases averted and shaded region indicates the 95% credible interval. **B**−**D** Assuming a time-varying force of infection, and decaying vaccine effectiveness, we predict a range between which the ratio of cases averted must fall (solid coloured regions, full opacity), for a 4 week (**B**), 26 week (**C**) and 52 week (**D**) dose spacing. The 95% credible intervals around the range is given by the shaded regions and dashed lines. Dotted horizontal lines indicate when the cases averted with the one-dose or two-dose strategy are equal, when the ratio of cases averted is above one, the one-dose strategy is optimal.

Of note, all these analyses assumed a low and constant force of infection (consistent with evidence of historic case numbers^25^). However, in an outbreak scenario the force of infection is likely to be higher and to also vary with time, perhaps increasing and then decreasing depending on the nature and duration of the outbreak. We now consider how the ratio of cases averted with the one- versus two-dose strategies might be influenced in the context of a time-varying force of infection and different levels of cumulative incidence over the 2 years following vaccine deployment. We first analyse the predicted cases averted using only the estimates of vaccine effectiveness from our previous meta-regression of real-world effectiveness studies (i.e. not accounting for waning immunity)^4^. In this scenario, the one dose strategy is predicted to be optimal except at very high incidence rates (cumulative incidence of >90% over 2 years) (Fig. 2A). In order to account for waning of vaccine effectiveness as well as a time-varying force of infection, one challenge is that the exact ratio of cases averted will depend on the exact force of infection over time. We wish to keep the time-varying force of infection unspecified to maintain generalisability of our results. Instead, we notice that we can determine limits on the ratio of cases averted such that - no matter what the exact time-varying force of infection—the true ratio of cases averted will be within these bounds (see Methods). Using these bounds and our estimates of vaccine effectiveness over time from our model based meta-analysis, we find that when vaccines are deployed 4 weeks after the first dose, the one-dose strategy is nearly always predicted to avert more cases than the two-dose strategy, regardless of the cumulative incidence (Fig. 2B). Taking a particular example, the one-dose strategy is predicted to avert at least 1.77-fold (95% CI: 1.58) more cases than the two dose strategy when the cumulative incidence is 20% or below. If vaccines are deployed later, at 26 weeks after first doses were administered, the one-dose strategy is still predicted to avert at least 1.58-fold (95% CI: 1.28) more cases than the two dose-strategy when the cumulative incidence is 20% or below, and similarly when administered 52 weeks after first doses (Fig. 2C, D). More generally, from this analysis, except when the cumulative incidence is above 75%, the one-dose strategy is predicted to avert more cases than the two-dose strategy over a 2 year time interval (Fig. 2).

### Considering heterogenous risk groups

**Fig. 3:**
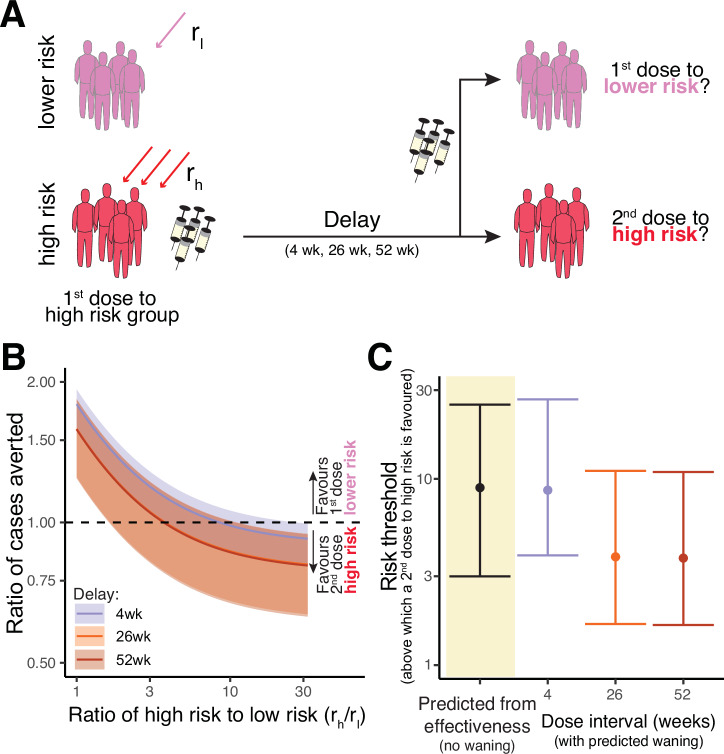
Predicting the effects of distributing vaccine doses between risk groups. **A** Schematic of the allocation scenario. We assume that a first dose of vaccine has been administered to a high-risk group, and at some later time more vaccine becomes available. These vaccines could either be allocated as second doses to the high-risk group, or administered as a first dose to a lower-risk group. **B** Depending on the ratio of risk in the high-risk and lower-risk groups (*x*-axis), then the predicted ratio of cases averted by the one-dose compared to the two-dose strategy (y-axis) will vary. This will also vary with the spacing between doses (colours), with longer delays since first dose in the high-risk group favouring the strategy of a second dose to the high-risk group. The horizontal dashed line shows a ratio of one (i.e. where we flip from favouring a one-dose approach to favouring a two-dose approach). Results are shown for prevention of mpox of any severity. **C** The risk threshold (y-axis) is the risk ratio at which the optimal strategy changes from favouring a one-dose to favouring a two-dose regimen. The risk threshold predicted from the vaccine effectiveness reported in clinical studies is on the left (black), and the threshold estimated from model-based meta-analysis (including vaccine waning for vaccine administered at 4, 26, or 52 weeks) is shown on the right. Analysis in (**B**) and (**C**) is based on a low and constant force of infection. Shaded regions and error bars indicate 95% credible intervals.

The above analyses only considered a population at homogeneous risk of infection. We see that in the case of a population at-risk and limited vaccine doses, giving as many people as possible a first dose of MVA-BN would avert more cases than giving half as many people two doses of MVA-BN. We next consider the possibility of some population subgroups being at greater risk of mpox. The latest data from the DRC highlights that both risk of mpox and the case-fatality ratios of clade I are higher in young children than older children and adults^2^. Therefore, it is important to consider when it might be optimal to deploy a second dose of MVA-BN to those subpopulations at higher risk, rather than to give a first dose to people at a lower risk for primary prevention of mpox. Returning to our simple scenario of a low and constant force of infection, we consider a scenario of two identifiable risk groups, one with high-risk, and one with lower risk of mpox infection. Initially, it is favourable to target the high-risk individuals with a first dose. However, when the majority of the high-risk individuals have already received a first dose of MVA-BN, we ask whether it is optimal to administer a second dose to the high-risk group or deliver a first dose to those in the lower risk group (Fig. 3A). Specifically, we consider how many times higher the risk would need to be in the high-risk subpopulation before it becomes optimal to target high-risk individuals with a second dose instead of targeting lower risk individuals with a first dose (which we refer to as a ‘risk-threshold’ above which the two-dose regimen is preferred).

**Fig. 4:**
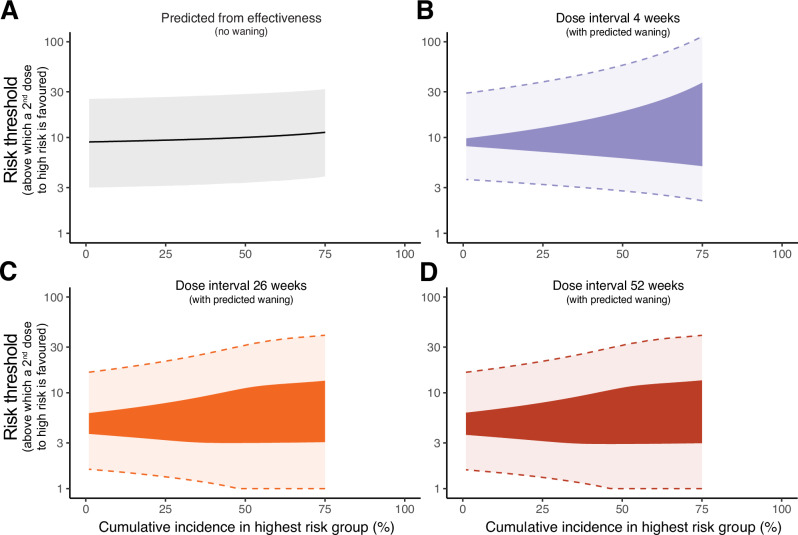
Predicting the risk threshold with a time-varying force of infection and different cumulative incidence. Here we compute the risk threshold, which is the fold-higher risk in a high-risk group compared to a lower risk group above which a second dose to higher risk individuals is the favoured strategy and below which a first dose to lower risk individuals is favoured. We assume there is an unknown time-varying force of infection, and a given cumulative incidence rate (in the high-risk group) over a 2 year time interval (x-axis). We first calculate this assuming no waning of vaccine effectiveness by using only the estimates of vaccine effectiveness from our meta-analysis of primary studies^4^ (**A**). The solid line is the estimated risk threshold for each cumulative incidence, and the shaded region is the credible interval around this estimate. We also use our previous model of predicted waning of vaccine effectiveness over time, and with different dose spacing^4^ to estimate the risk threshold (**B**−**D**). In these cases, the exact risk threshold will depend on the exact time-varying force of infection, which we have chosen to leave unspecified. Thus instead of computing an exact risk-threshold we compute a range within which the actual risk threshold must be contained regardless of the exact time-varying force of infection. This range is given by the coloured region (full opacity). The credible intervals of this range are indicated by the shaded region and dashed lines. Note that above a cumulative incidence of ~75% there is no single risk threshold, thus we have truncated our estimates here.

Using only the vaccine effectiveness from our meta-analysis of real-world data, we estimate that unless the high-risk group is at least 9.0-fold (95% CI: 3.0-25) more likely to be infected than the lower risk group, it is preferable to administer any additional vaccines as first doses to the lower risk group rather than administering second doses (following the 4 week dosing interval) to the high-risk group. If we take into account waning immunity and protection over the first 2 years after deployment, we see a similar risk-threshold for when we should favour a two-dose regimen, if deployment is occurring 4 week after peoples first doses (Fig. 3B). If additional vaccines only become available at 26 or 52 weeks after the first dose, the risk-threshold is reduced to 3.8-fold (CI: 1.6-11) because of waning immunity after the first dose and a higher immune response after a delayed second dose (Fig. 3C) (using the assumption that a second dose at 26 or 52 weeks gives the same peak protection as that predicted after a boost at 2 years, Fig. 1B, which is a conservative assumption, as it tends to decrease the risk-threshold). Finally, removing the assumption of low and constant force of infection (i.e. allowing for higher and time-varying force of infection), the results remain very similar (Fig. 4). For example, when the 2-year cumulative incidence rate remains below 20%, we see that when vaccines are deployed at 4, 26 or 52 weeks, the one-dose strategy is predicted to be optimal as long as the high-risk group has no more than an 7.3-fold (2.5% one-sided CI: 3.3), 3.3-fold (CI: 1.4) and 3.3-fold (CI: 1.4) higher risk than the lower risk group, respectively. These risk thresholds indicate the situations where no matter the exact form of the time varying force of infection the one-dose strategy is optimal. Above this threshold, it is possible that a one dose strategy may still be preferable, but would depend on the exact force of infection over time (dark shaded region, full opacity, in Fig. 4). However, when the risk in the high-risk group is very high (at least 12 (97.5% one-sided CI: 36), 7.4 (CI:20) and 7.3-fold (CI: 20) higher risk of mpox than the lower risk group), then a two-dose strategy is predicted to be optimal, regardless of the exact form of the time varying force of infection.

### Averting severe outcomes

In the analyses above we only considered the optimal strategy to avoid the most mpox cases of any severity (mild, moderate or severe). However, case-fatality ratios in children under 5 years of age have been reported at 3.2 times higher than in adults and children older than 15 years^2^. These observations are based on very limited epidemiological data and may suffer from a range of confounding^27^. However, if the goal of vaccination were to reduce severe mpox (rather than any clinical mpox infection), this raises the question of whether we should favour deploying limited vaccine supplies as a one- or two-dose regimen, and under what conditions we should target high-risk groups for two-dose vaccination?

Unfortunately, there is very limited data available on vaccine effectiveness in preventing severe mpox infection (and none for clade I infection). However, a comprehensive meta-analysis of studies of individuals with breakthrough mpox clade IIb infection suggested a 66.6% (95% CI: 55-78%) vaccine effectiveness against progression from mild infection to hospitalisation^5^, and there appears similar vaccine effectiveness in preventing progression to severe mpox after either one or two doses of MVA-BN. Assuming that one dose MVA-BN has 74% effectiveness at preventing infection and two doses of MVA-BN has 82% effectiveness, and assuming both provide 66% protection against progressing to severe outcomes, we can estimate from this that the vaccine effectiveness at preventing severe infections that lead to hospitalisation is around 91% for a one-dose, and 94% for a two-dose regimen (see methods). Given the absence of clade specific vaccine effectiveness data against severe mpox after 1 and 2 doses of MVA-BN in the populations at-risk from each clade (e.g. children for clade Ia), the modelling of vaccine impact on severe mpox has a number of additional caveats. Nonetheless given the available data and these assumptions we sought to provide a prediction on optimal strategies for preventing severe mpox.

**Fig. 5:**
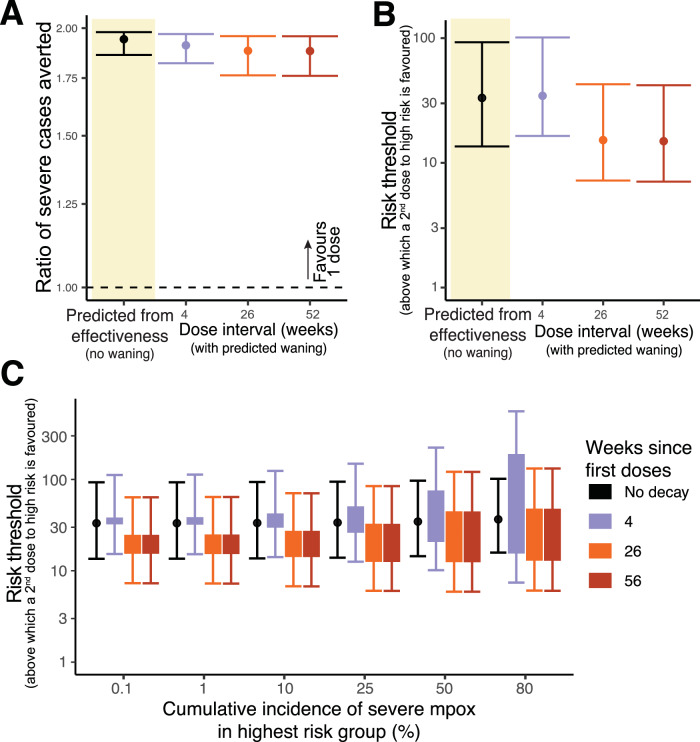
Comparison of vaccine strategies to prevent severe mpox cases. **A** The ratio of severe cases averted by using a one-dose strategy compared to a two-dose strategy - based on the vaccine effectiveness observed in the clinical studies^4^, with no waning of immunity (left, black) or predicted (when the doses are spaced at 4, 26, or 52 weeks) using estimates of immune waning from a model-based meta-analysis^4^ (right). **B** The risk threshold (the ratio of risk in the high-risk group compared to the low risk) that favours the switch from the one-dose to the two-dose regimen for severe mpox. The risk threshold predicted from the vaccine effectiveness reported in clinical studies (left, black), and the model-based meta-analysis for vaccine administered at 4, 26, or 52 weeks (right). Panels (**A**, **B**) assume a low and relatively constant force of infection. **C** Generalising to consider a variable force of infection and cumulative incidence of severe mpox, we predict the risk threshold above which administering second doses to a high-risk group will avert more cases than administering a first dose to a lower risk group. The predicted risk threshold in the case of a constant (non-decaying) vaccine effectiveness is given by a black dot. The rectangular bars indicate the range between which the risk threshold must fall when waning of vaccine effectiveness is taken into account and with different dose-spacing. Error bars indicate 95% credible intervals.

In the case of a low and constant force of infection, using only these estimates of vaccine effectiveness from the meta-analysis of real-world studies, we can predict that, for a homogenous population, administering additional vaccines as a first dose to naïve individuals will prevent 1.94-fold (CI: 1.86-1.98) more cases of severe mpox than the alternative (Fig. 5A). We observe similar results when accounting for waning of protection (Fig. 5A). Thus, as was the case when looking at averting mpox cases of any severity (Fig. 1E), the one-dose strategy is predicted to be optimal if the goal of vaccination is to maximise protection from severe mpox.

As we did above for mpox cases of any severity, we can also analyse the situation where there are identifiable subgroups that are at higher risk of severe infection. Again, here we consider a scenario where the high-risk group has already received one dose of vaccine, and we have the choice of deploying additional vaccine doses as second doses to the high-risk group, or as first doses to a low-risk group. Using only the data from the real-world studies of vaccine effectiveness (without waning), we predict that unless the high-risk group were at greater than 33-fold (CI:14-93) higher risk of severe infection than the low-risk group, we should favour administering one dose of the vaccine to as many people as possible rather than giving a second dose to the high-risk population (Fig. 5B). The risk threshold is very similar when accounting for the predicted waning of vaccine effectiveness over time (Fig. 5B). That is, applying our predictions of waning vaccine effectiveness^4^, we predict that early after vaccination, giving a first dose to more people would be favoured unless the high-risk group had at least 34-fold (CI:16-101) times higher risk of severe infection compared to the low-risk group (Fig. 5B). If a second dose is delayed to 26 or 52 weeks after the first dose (again using the conservative assumption that a second dose at 26 or 52 weeks gives the same peak protection as it would at 2 years), the one dose strategy will remain favoured unless the high-risk group has more than a 15-fold (CI: 7-42) higher risk of severe infection compared to the lower risk group (Fig. 5B). Again, considering the same scenarios but under the more general case of a time varying force of infection, we see the results are very similar, even at high overall incidence rates of severe mpox (Fig. 5C).

## Discussion

In the context of adequate vaccine availability, delivering the recommended two dose MVA-BN schedule to as many people as possible can achieve rapid population protection. Here we do not advocate for changes to the recommended vaccine schedule generally, or delaying the second dose of MVA-BN as a standard approach. However, in the context of the current public health emergency, there are limited vaccine supplies available in the countries most in need. We address questions about the optimal deployment of these limited vaccine resources. Here we find that in a population (or subpopulation) of homogenous risk, deploying limited vaccine supplies as single doses to as many people as possible is virtually always favoured. The implication of this is that, in the presence of high-risk subpopulations, it is predicted to be optimal to target as many high-risk individuals as possible with a first dose before considering second doses. However, once the high-risk population has largely received a first dose, a question arises as to whether to deploy second doses to these high-risk individuals or deploy vaccines as first doses to lower risk populations. Whether it will be optimal to focus efforts on the high-risk population or spread doses into lower risk populations depends on (i) how many times higher the risk of mpox is in the high-risk populations compared with the lower risk population, and (ii) how long ago the high-risk individuals received their first dose. Interestingly, the optimal strategy has a relatively limited dependence on the cumulative incidence when this is below 75% (Fig. 2). The risk threshold (below which it is optimal to deploy first doses to lower risk individuals instead of second doses to higher risk individuals) is relatively constant as the cumulative incidence increases, but the uncertainty as to which strategy is optimal tends to increase (due to wide bounds on the risk threshold and large credible intervals, Fig. 4). The preference for one dose is more pronounced when considering prevention of severe mpox, largely because the vaccine effectiveness against this outcome is estimated to be very high even after one dose. The risk threshold for averting more severe mpox is around 12-fold (i.e. the one dose strategy is favoured unless the high-risk group has greater than 12-fold higher risk after which point it is not clear which strategy will be favoured, Fig. 5C).

Here we have considered deploying available vaccines as one dose regimens as a means of extending the coverage of a limited supply of MVA-BN vaccines. However, there are other important considerations and approaches we have not considered explicitly in our analysis. Firstly, our analysis does could not account for many of the most critical determinants of vaccine rollout, such as local constraints on health resourcing, social acceptability and trust of health interventions, and the systems for vaccine tracking and follow-up. Secondly, our analysis of risk groups assumes we can have an accurate understanding of who is at-risk, and the relative risk between different subgroups. This may be more or less clear in different settings and depends on the distinct epidemiology of different mpox clades. For example, in Kenya all detected clade Ib cases have occurred along one transport corridor and a half of those have occurred in truck drivers or those in contact with truck drivers including sex workers^28^. This suggests a very concentrated risk group where targeting with two doses may be optimal, to the extent this is practicable given local constraints. In contrast, in western and central DRC, where clade Ia mpox is endemic and consistent sporadic outbreaks are known to occur^29^, the risk profile within an area may be more complex, flatter and/or unclear. In this case, maximising the number of people with a single dose may be a valuable option. Thirdly, we have only considered vaccine deployment as pre-exposure prophylaxis to high-risk groups. However, strategies such as ring vaccination or vaccinating contacts of known mpox cases may also be beneficial as they are a means of ensuring vaccines reach those who likely have a very high risk of contracting mpox. Lower vaccine effectiveness might be expected when targeting contacts of cases, since current estimates for mpox vaccine effectiveness when used as post-exposure prophylaxis have high uncertainty, but may be quite low^5^. Finally, altering the route of administration from the recommended subcutaneous injection to an intradermal injection should also be considered in the context of limited vaccine supply. It has been shown that intradermal administration of one-fifth of the standard subcutaneous dose achieves similar antibody titres^30^. Further, the intradermal route was successfully used to extend MVA-BN vaccine coverage during the global epidemic ongoing since 2022, and vaccine effectiveness following the standard subcutaneous and the one-fifth dose intradermal route achieved similar vaccine effectiveness^5^. Therefore, this analysis must be viewed in light of other important factors as a useful tool to aid local and national policy makers.

Our analysis considers two different methods in which to compare the effectiveness of one- and two-dose regimens. The first approach uses the observed vaccine effectiveness derived from a meta-analysis of clinical studies (Figs. 1E, 2C). The second approach assumes vaccine effectiveness wanes according to the decline in antibody titres^4^. Each of these approaches come with a number of caveats and limitations. In the first approach, we use only the estimates of vaccine effectiveness from meta-analyses of real-world effectiveness studies to compare the ratio of cases averted under the different vaccination strategies. A strength of this approach is that it makes no assumptions about the duration of protection (for which there is currently very limited clinical data). However, as a result this approach implicitly assumes vaccine effectiveness does not wane. The second approach involves analysis of the relationship between vaccinia-binding antibody titres and vaccine protection, along with analysis of antibody boosting and waning antibody levels to predict vaccine effectiveness at different times^4^. This is of course significantly less direct than the approach described above and relies on the assumption that vaccinia-binding antibody levels are predictive of vaccine effectiveness against mpox over time, and that antibody titres and immunity will wane in a similar way in the populations at risk of mpox. Although there is limited data to support vaccinia-binding as a correlate of protection against mpox^4^, non-inferiority of serological responses to vaccination between MVA-BN and ACAM-2000 were used to inform the FDA approval of MVA-BN^18^. A functional measure, such as neutralising antibodies against mpox, is likely a more direct correlate of protection. Further data to support a correlate of protection for mpox vaccination are urgently needed to support vaccine deployment decisions. Other limitations of the vaccine effectiveness modelling approach are outlined in detail in the original study^4^. Rather than relying on predictions of the vaccine effectiveness over time from the decay in antibody responses, further data on real world effectiveness of vaccination out to 1–2 years post MVA-BN vaccination (with either one or two doses) would be very informative. Fortunately, this data is likely available, or soon to be available, in many settings given vaccination during the 2022/2023 clade IIb outbreaks and ongoing clade IIb transmission globally. Further, much of the data used to inform our analysis is based on vaccine effectiveness against clade IIb from real world effectiveness studies conducted outside of Africa during the current global clade IIb pandemic^4^. An inherent assumption is that protection against clade Ia and Ib mpox is similar to clade IIb mpox. This assumption is neither supported nor contradicted by existing data (although one study shows similar binding antibody titres between clade Ib and IIb among vaccinated individuals^31^). Clearly further studies of vaccine effectiveness against clade Ia and Ib are urgently needed. Further, studies to validate antibody levels as a predictor of vaccine effectiveness against mpox and to investigate antibodies, cell-mediated immunity and protection over time in the context of clade I virus would be valuable to aid in policy decisions for mpox vaccine deployment.

We analyse the different vaccination strategies under two infection scenarios. Firstly, assuming low and constant force of infection (which is largely consistent with zoonotic transmission levels observed in the DRC since 2010-2018^25^). Secondly, we consider a situation of time varying incidence rate and higher levels of cumulative incidence over the time following vaccination (here we consider a 2 year period). We note that in modelling a time-varying force of infection we have opted to keep our conclusions as generalisable as possible and thus have chosen not to explicitly define the time-varying force of infection. Instead, we have inferred bounds on the ratio of cases with one strategy or another, rather than calculate exact values. However, this means that between these intervals we are unable to predict which strategy will be optimal.

All our analysis approaches assume that vaccine supply is low compared to the total susceptible population, such that the deployment strategies do not impact the force of infection. Further, we do not consider time to epidemic control, where the delay between dose 1 and dose 2 would be influential. Instead, we consider only cases averted or severe cases averted with each strategy, which may not be the only metric of relevance, especially in a very high incidence scenario, where for example reducing load on health care systems may be an important consideration. Also, all our analyses rely on the results of our meta-analysis of real-world effectiveness studies^4^. There are now three systematic reviews of MVA-BN vaccine effectiveness^4–6^, which give similar estimates of one and two-dose vaccine effectiveness in the context of clade IIb infection. However, the underlying real-world studies included in these meta-analyses each have considerable limitations^4,5^.

Our analysis of vaccine effectiveness against severe mpox has additional caveats. There is very limited data on vaccine effectiveness against severe mpox, even for the globally circulating clade IIb, and we base our analysis of severe protection on a single meta-analysis of vaccine effectiveness against progression from symptomatic to severe clade IIb infection^5^. Again, we assume vaccine effectiveness against progression to severe mpox is similar for clade I as for clade IIb. This was necessary since no data exists on the protection against severity of clade I mpox. Since disease severity appears to differ by clade^32^, it is critical to validate if vaccine effectiveness against severe mpox differs by clade. Vaccine effectiveness and duration of immunity may differ for groups at risk of severe mpox such as children, people with HIV, or immunocompromised individuals^33^ (although recent evidence suggests similar effectiveness in people living with HIV)^34^. Currently there is minimal data available assessing efficacy and immunogenicity in these risk groups. Studies are urgently needed to understand vaccine immunogenicity and effectiveness across different at-risk populations and for different clades in order to guide rational vaccine deployment.

Notwithstanding the many limitations of the available data, this analysis suggests that where limited supplies of MVA-BN vaccine are available, deploying them as a single dose regimen to as many people as possible will usually be the most effective strategy to reduce mpox cases. However, providing second doses to high-risk populations may be favoured if the risk-ratio of the high-risk to the general population is sufficiently high. Delaying second doses in the context of limited vaccine supply is not a novel approach. During the COVID-19 pandemic limited supply of the AstraZeneca vaccine in the UK led to increased dosing intervals^35^, and this was accompanied by higher vaccine efficacy than reported with the original (4 week) dosing schedule^36^. These conclusions do not advocate for a change in the recommended vaccine schedule for MVA-BN, or for health authorities to ignore this schedule. Vaccine should be deployed with the intention of giving a second dose at 4 weeks or as soon as possible. However, limited vaccine availability may at times dictate that allocating limited vaccine supplies as a second dose to those already vaccinated is not an efficient strategy to maximise population protection.

## Methods

The analysis and modelling of publicly available de-identified clinical trial data was approved under the UNSW Sydney Human Research Ethics Committee (approval HC200242).

### Modelling the averted cases

Here we seek to compare two vaccine deployment strategies. We consider the scenario in which some individuals have already received a first dose of vaccine, and at some later time more vaccine doses become available that can either be deployed as second doses to those already vaccinated with one dose or deployed as first doses to naïve individuals. We assume the number of additional vaccines available, $D$, is much less than the vulnerable population, $P$, and that $n$, is the population that has received a first dose $s$ days earlier. It follows that the number of vaccines that can be given as second doses (for which a deployment decision is required) is given by $N=\min \left(n,D\right)$. By postulating that the number of vaccines is small compared to the total at risk population, we assume that the vaccine deployment decision does not affect the force of infection. Therefore, for a population of $2N$ individuals, $N$ of whom have been previously vaccinated $s$ days earlier (where $s\ge 28$), and $N$ of whom are unvaccinated, we can either deploy the $N$ vaccines as second doses into the already vaccinated population or as first doses in the unvaccinated population.

To compare these different vaccine strategies, we will calculate the ratio of cases averted between the two competing strategies, using the estimates for vaccine effectiveness of one- and two-dose regimens reported from our previous meta-analysis of real-world effectiveness studies^4^. The primary studies in our meta-analysis typically defined vaccine effectiveness as one minus the odds ratio, either of (1) cases in unvaccinated versus vaccinated individuals or (2) vaccination status in cases and controls, depending on the study design^4^.

To model this, we first considered a low and constant force of infection (infection rate), $r$, within the population of concern, which is representative of, for example, low levels of zoonotic transmission^25^, combined with waning vaccine effectiveness. In this case, the number of cases averted, CA, by a given vaccine strategy when deployed in a group of individuals (compared to an equivalent unvaccinated group) is given by,

1 $$\begin{array}{c}{\rm{CA}}=\,{\rm{Vaccine\; Effectiveness}}\times {\rm{Number\; of\; people\; vaccinated}}\times {\rm{Force\; of\; infection}}.\,\end{array}$$

If the $N$ doses in question are administered to $N$ unvaccinated individuals, it follows that the expected number of cases averted (compared to a population of $2N$ unvaccinated individuals) is given by the cases averted due to the prior vaccination $s$ days earlier of the $N$ originally vaccinated individuals plus those averted in the $N$ newly vaccinated individuals,

2 $$\begin{array}{c}C{A}_{1}\left(t\right)={\int }_{0}^{t}V{E}_{1}\left(x+s\right){dx}\times N\times r+{\int }_{0}^{t}V{E}_{1}\left(x\right){dx}\times N\times r,\end{array}$$

where, $C{A}_{1}\left(t\right),$ is the cumulative number of cases averted by time $t$ (in days), ${\int }_{0}^{t}V{E}_{1}\left(x\right){dx}$ is the cumulative vaccine effectiveness of one dose of MVA-BN over time $t,$ and $r$ is the infection rate (i.e. force of infection). Note that the term $V{E}_{1}(x+s)$ captures those who were vaccinated with one dose *s* days before the current batch of vaccines were deployed, and thus the effectiveness from this dose will have waned for $s$ days before our decision point.

Similarly, if these $N$ doses are instead administered as second doses, the cumulative number of cases averted by time $t$ under the two-dose strategy (compared to a population of $2N$ unvaccinated individuals), $C{A}_{2}\left(t\right)$, is given by,

3 $$\begin{array}{c}C{A}_{2}\left(t\right)={\int }_{0}^{t}V{E}_{2,s}\left(x\right){dx}\times N\times r.\end{array}$$

where ${\int }_{0}^{t}V{E}_{2,s}\left(x\right){dx}$ is the cumulative vaccine effectiveness of two doses of MVA-BN over time $t$, and $s$ is the interval between the two administered doses. This contains only one term because there is a population of $N$ individuals who would then not receive any vaccine (no protection) and who do not contribute to the number of cases averted. The number of cases averted is dependant only on the population of $N$ individuals who would have 2 doses.

Thus, we can define the ratio of cases averted by time $t$ as,

4 $$\begin{array}{c}{RCA}\left(t\right)=\frac{C{A}_{1}\left(t\right)}{C{A}_{2}\left(t\right)}=\frac{{\int }_{0}^{t}V{E}_{1}\left(x\right){dx}+{\int }_{0}^{t}V{E}_{1}\left(x+s\right){dx}}{{\int }_{0}^{t}V{E}_{2,s}\left(x\right){dx}}.\end{array}$$

This ratio of cases averted by time *t* is equivalent to the ratio of the average vaccine effectiveness in the population between the time of administration and time $t$. To calculate the *RCA* at a given time $t$, we can either assume vaccine effectiveness is not waning, or we require estimates of vaccine effectiveness over time. In both cases we use the results from our fitted model analysing vaccine effectiveness data and predicting the rate of waning of vaccine effectiveness with time from our previously published model-based meta-analysis of real world-effectiveness data^4^. The results from our meta-analysis^4^ are used calculate the ratio in Eq. (4), and the credible intervals around this ratio (details in posterior sampling section).

### Incorporating groups with different risk of mpox or severe mpox

We can further extend our above model to consider vaccinating two subpopulations that have different risks of becoming infected (different forces of infection). In this scenario we consider a high-risk group in which everyone has received a first dose of the vaccine. We then consider a scenario where additional vaccine doses become available and we must decide whether to deploy them as second doses for individuals in the already vaccinated high-risk population, or as first doses to individuals in a lower-risk population. We can estimate a ‘risk threshold’, which is how many times higher the risk of infection must be in the high-risk population in order that targeting the high-risk group with a second dose will be the favoured scenario. Below this ‘risk threshold’ it will still be optimal to use available doses to vaccinate lower risk individuals with first doses. When considering different risk groups, whilst assuming a low and constant force of infection, the cases averted by administering a single dose are given by,

5 $$\begin{array}{c}C{A}_{1}\left(t\right)={\int }_{0}^{t}V{E}_{1}\left(x\right){dx}\times N\times {r}_{l}+{\int }_{0}^{t}V{E}_{1}\left(x+s\right){dx}\times N\times {r}_{h},\end{array}$$

where, ${r}_{h}$ and ${r}_{l}$ are the rate of infection in the high and low risk groups, respectively. Similarly, the cases averted in the two-dose strategy are given by,

6 $$\begin{array}{c}C{A}_{2}\left(t\right)={\int }_{0}^{t}V{E}_{2,s}\left(x\right){dx}\times N\times {r}_{h}.\end{array}$$

Thus, we can calculate the ratio of cases averted in a mixed population over time, ${{RCA}}_{m}\left(t\right)$, as

7 $$\begin{array}{c}{{RCA}}_{m}\left(t\right)=\frac{\left({\int }_{0}^{t}V{E}_{1}\left(x\right){dx}\times 1/\left({r}_{h}/{r}_{l}\right)\right)+{\int }_{0}^{t}V{E}_{1}\left(x+s\right){dx}}{{\int }_{0}^{t}V{E}_{2,s}\left(x\right){dx}},\end{array}$$

We note that if the two populations have equal risk (${r}_{h}/{r}_{l}=1$), then Eq. (7) is identical to Eq. (4). We define the risk threshold, ${r}_{c}$, to be the ratio such that when ${r}_{h}/{r}_{l} > {r}_{c}$, then more cases are averted by administering second doses to the high-risk group. This risk threshold can be estimated by solving the equation ${{RCA}}_{m}=1$, which occurs when

8 $$\begin{array}{c}{r}_{c}=\frac{{\int }_{0}^{t}V{E}_{1}\left(x\right){dx}}{{\int }_{0}^{t}V{E}_{2,s}\left(x\right){dx}-{\int }_{0}^{t}V{E}_{1}\left(x+s\right){dx}}.\end{array}$$

The risk threshold, ${r}_{c},$ can be calculated across any time period. We use a period of 2 years in our analysis.

By combining Eq. (7) with real-world effectiveness estimates we can estimate the RCA in a mixed population with a constant vaccine efficacy and a low and constant force of infection. In this case Eq. (7) simplifies to,

9 $$\begin{array}{c}{{RCA}}_{m}\,=\frac{V{E}_{1}\frac{{r}_{l}}{{r}_{h}}+V{E}_{1}}{V{E}_{2}}.\end{array}$$

This relationship depends on the infection risk ratio between high-risk and low-risk individuals. For the special case of constant vaccine efficacy, the risk threshold simplifies to

10 $$\begin{array}{c}{r}_{c}=\frac{V{E}_{1}}{V{E}_{2}-V{E}_{1}}.\end{array}$$

Thus, under these simplified assumptions, more cases will be averted by a one dose strategy, except when $\frac{{r}_{h}}{{r}_{l}} > \frac{V{E}_{1}}{V{E}_{2}-V{E}_{1}}$.

### Generalising the comparison of one and two dose strategies when the force of infection is not constant with time

Here we derive a model that captures the scenario when the force of infection, $r$, varies with time, so that $r$ is given by $r\left(t\right)$.

We consider an unvaccinated susceptible population, ${S}_{u}\left(t\right)$, with a varying rate of exposure to infection over time. The number of susceptible unvaccinated individuals over time, $t$, ${S}_{u}\left(t\right)$, is described by

11 $$\begin{array}{c}\frac{d{S}_{u}}{{dt}}=-r\left(t\right){S}_{u},\,\end{array}$$

where $r\left(t\right)$ is the infection rate at time, $t$. Here we ignore effects of individuals becoming susceptible again (i.e. we assume infected individuals acquire a high level of immunity for the duration of our analysis). We additionally assume that the susceptible vaccinated individuals, ${S}_{v}\left(t\right)$, have a force of infection that is reduced by one minus the vaccine effectiveness (VE), and so the number of vaccinated, susceptible individuals, ${S}_{v}\left(t\right)$, is described by

12 $$\begin{array}{c}\frac{d{S}_{v}}{{dt}}=-\left(1-{VE}\left(t\right)\right)r\left(t\right){S}_{v}.\end{array}$$

Solving Eqs. (11) and (12) we find that the number of unvaccinated and vaccinated susceptible individuals at a time $t$ are given by

13 $$\begin{array}{c}{S}_{u}\left(t\right)={S}_{u}\left(0\right){e}^{-{\mathop{\int }\nolimits_{\!0}^{t}}r\left(\lambda \right)d\lambda }\end{array}$$

and

14 $$\begin{array}{c}{S}_{v}\left(t\right)={S}_{v}\left(0\right){e}^{-{{\mathop{\int }\nolimits_{\!0}^{t}}}r\left(\lambda \right)\left(1-{VE}\left(\lambda \right)\right)d\lambda }\end{array}$$

respectively.

The corresponding cumulative number of cases in unvaccinated and vaccinated individuals that have occurred by time $t$ are given by

15 $$\begin{array}{c}{I}_{u}\left(t\right)={S}_{v}\left(0\right)\left(1-{e}^{-{{\mathop{\int }\nolimits_{\!0}^{t}}}r\left(\lambda \right)d\lambda }\right)\end{array}$$

and

16 $${I}_{v}\left(t\right)={S}_{v}\left(0\right)\left(1-{e}^{-{{\mathop{\int }\nolimits_{\!0}^{t}}}r\left(\lambda \right)\left(1-{VE}\left(\lambda \right)\right)d\lambda }\right),$$

respectively.

Under these assumptions, the ratio of cases averted in a homogeneous population is given by,

17 $${RC}{A}_{h}\left(t\right)=\frac{C{A}_{1}\left(t\right)}{C{A}_{2}\left(t\right)}=\frac{\left(2-{e}^{-\mathop{\int}\nolimits_{\!0}^{t}r\left(\lambda \right)\left(1-V{E}_{1}\left(\lambda \right)\right)d\lambda }-{e}^{-\mathop{\int }\nolimits_{\!0}^{t}r\left(\lambda \right)\left(1-V{E}_{1}\left(\lambda +s\right)\right)d\lambda }\right)}{\left(2-{e}^{-{\mathop{\int}\nolimits_{\!0}^{t}}r\left(\lambda \right)d\lambda }-{e}^{-\mathop{\int}\nolimits_{\!0}^{t}r\left(\lambda \right)\left(1-V{E}_{2,s}\left(\lambda \right)\right)d\lambda }\right)},$$

where $C{A}_{1}(t)$ and $C{A}_{2}(t)$ are the cases averted in the one and two dose scenarios, respectively. As previously, if we assume a constant vaccine efficacy over time (allowing us to use the real-world estimates for vaccine effectiveness from our previous study^4^) Eq. (17) simplifies to

18 $$\begin{array}{c}{RC}{A}_{h}\left(t\right)=\frac{2{\alpha }_{t}-2{\alpha }_{t}^{1-V{E}_{1}}}{{\alpha }_{t}-{\alpha }_{t}^{1-V{E}_{2}}},\end{array}$$

where, ${\alpha }_{t}={e}^{-\mathop{\int }\nolimits_{\!0}^{t}r\left(\lambda \right)d\lambda }$. Once again, we can consider the case of two subpopulations of individuals, one with a higher and the other with a lower risk of mpox (as in Eqs. (5)-(7)), but this time under a non-constant force of infection. Assuming that higher risk individuals have a force of infection at time $t$ given by ${r}_{h}\left(t\right)$, and lower risk individuals have a force of infection at time $t$ given by ${r}_{l}\left(t\right)$, and that the fold higher risk for the high-risk population compared to a lower risk population, $F$, is constant over time, i.e. $F=\frac{{r}_{h}\left(t\right)}{{r}_{l}\left(t\right)}$, for all $t$. It follows that the ratio of cases averted in the generalised scenario is given by:

19 $$\begin{array}{c}{RC}{A}_{m}\left(t\right)=\frac{{a}_{t}^{\frac{1}{F}}+{a}_{t}-{a}_{t}^{\left(\frac{1}{F}\right)\mathop{\int }\nolimits_{\!0}^{t}h\left(\lambda \right)\left(1-V{E}_{1}\left(\lambda \right)\right)d\lambda }-{a}_{t}^{\mathop{\int }\nolimits_{\!0}^{t}h\left(\lambda \right)\left(1-V{E}_{1}\left(\lambda +s\right)\right)d\lambda }}{{a}_{t}-{a}_{t}^{\mathop{\int }\nolimits_{\!0}^{t}h\left(\lambda \right)\left(1-V{E}_{2,s}\left(\lambda \right)\right)d\lambda }},\,\end{array}$$

where, ${a}_{t}={e}^{-{\int }_{0}^{t}{r}_{h}\left(\lambda \right)d\lambda }$, represents the proportion of high-risk susceptible individuals in an unvaccinated population who would remain uninfected by time $t$ (given the force of infection ${r}_{h}\left(t\right)$) and,

20 $$\begin{array}{c}h\left(\lambda \right)=\frac{{r}_{l}\left(\lambda \right)}{{\int }_{0}^{t}{r}_{l}\left(x\right){dx}},\end{array}$$

represents the force of infection at time $\lambda \in \left[0,t\right]$ normalised by the cumulative incidence over the entire interval in the low-risk population $\left[0,t\right]$.

Whilst Eqs. (17) and (19) will allow us to calculate the exact ratio of cases averted for a given risk ratio, $F$, cumulative incidence in the high-risk group (i.e. $1-{a}_{t}$), and vaccine effectiveness functions, it will also depend on the exact time varying distribution in the force of infection, $h\left(\lambda \right)$. However, it is also possible to derive bounds on the ratio of cases averted which do not rely on this exact force of infection function. These bounds are given by:

21 $$\begin{array}{c}\begin{array}{c}{RC}{A}_{m}\left(t\right)\le \min \left(\frac{{a}_{t}^{\frac{1}{F}}+{a}_{t}-{a}_{t}^{\left(\frac{1}{F}\right)}{a}_{t}^{-\left(\frac{1}{F}\right)V{E}_{1}\left(t\right)}-{a}_{t}\,{a}_{t}^{-V{E}_{1}\left(t+s\right)}}{{a}_{t}-{a}_{t}\,{a}_{t}^{-V{E}_{2,s}\left(0\right)}}\right.,\,\\ \left.\mathop{\max }\limits_{\mu \in \left[0,t\right]}\frac{\left(\frac{{a}_{t}^{\left(\frac{1}{F}\right)-1}}{F}\right)V{E}_{1}\left(\mu \right)+V{E}_{1}\left(\mu +s\right)}{V{E}_{2,s}\left(\mu \right)}\right)\end{array}\end{array}$$

and

22 $$\begin{array}{c}\begin{array}{c}{RC}{A}_{m}\left(t\right)\ge \max \left(\frac{{a}_{t}^{\frac{1}{F}}+{a}_{t}-{a}_{t}^{\left(\frac{1}{F}\right)}{a}_{t}^{-\left(\frac{1}{F}\right)V{E}_{1}\left(0\right)}-{a}_{t}\,{a}_{t}^{-V{E}_{1}\left(s\right)}}{a-a\,{a}^{-V{E}_{2,s}\left(t\right)}},\right.\\ \left.\mathop{\min }\limits_{\theta \in \left[0,t\right]}\left(\frac{{a}_{t}^{\left(\frac{1}{F}\right)-1}}{F}\right)\frac{V{E}_{1}\left(\theta \right)}{V{E}_{2,s}\left(\theta \right)}{a}_{t}^{\mathop{\max }\limits_{\lambda \in \left[0,t\right]}\left(V{E}_{2,s}\left(\lambda \right)-\frac{1}{F}V{E}_{1}\left(\lambda \right)\right)}+\frac{V{E}_{1}\left(\theta +s\right)}{V{E}_{2,s}\left(\theta \right)}{a}_{t}^{\mathop{\max }\limits_{\lambda \in \left[0,t\right]}\left(V{E}_{2,s}\left(\lambda \right)-V{E}_{1}\left(\lambda +s\right)\right)}\right).\end{array}\end{array}$$

A derivation of equations and inequalities (17)-(22) are presented in the supplementary information.

Once again, for the special case when we assume that vaccine effectiveness is constant, we can simplify Eq. (19). In this scenario the ratio of cases averted by the two strategies in Eq. (19) simplifies to

23 $$\begin{array}{c}{RC}{A}_{m}\left(t\right)=\frac{{a}_{t}^{\frac{1}{F}}+{a}_{t}-{a}_{t}^{\frac{1}{F}\left(1-V{E}_{1}\right)}-{a}_{t}^{1-V{E}_{1}}}{{a}_{t}-{a}_{t}^{1-V{E}_{2}}}.\end{array}$$

Note that this simplification relies on the fact that ${\int }_{0}^{t}h\left(\lambda \right)d\lambda =1$. We can then compute the ratio of cases averted for a given ${a}_{t}$ and $F$, using only the estimates of vaccine effectiveness after one and two doses from our meta-analysis of real world effectiveness studies^4^.

### Calculating the risk threshold with variable force of infection

We have previously calculated the critical risk threshold for a constant force of infection (Eq. (8)). We would now also like to calculate this for a variable force of infection. We remember that the critical risk threshold is the value, ${r}_{c}$, such that when $F > {r}_{c}$ a two-dose strategy is favoured (i.e. ${RC}{A}_{m}\left(t\right) < 1$), and when $F < {r}_{c}$ a one dose strategy is favoured, (i.e. ${RC}{A}_{m}\left(t\right) > 1$), and the risk threshold is determined by finding the value ${r}_{c}$ such that when $F={r}_{c}$, then ${RC}{A}_{m}\left(t\right)=1$. When both vaccine efficacy and the force of infection are non-constant, it is not possible to directly estimate ${r}_{c}$ as we did previously (in Eq. (8)), and instead we must use the bounds in Eqs. (21) and (22) to provide an upper and lower bound on the risk threshold. We therefore evaluate the upper and lower bounds of the critical risk threshold, (${r}_{c}^{u}$ and ${r}_{c}^{l}$ respectively) by solving equations where the upper and lower bounds in Eqs. (21) and (22) respectively are equal to 1. These were solved numerically using nleqslv package^37^ in R version 4.4.1^38^. When the force of infection was high, there were multiple values for the fold risk, F, that resulted ${RC}{A}_{m}(t)=1$, therefore, in these regions we do not define a critical risk threshold.

### Calculating VE against severe mpox

Vaccine effectiveness estimates from our previous meta-analysis of real-world effectiveness studies were all focused on protection against mpox of any severity^4^. However, here we wished to estimate vaccine effectiveness against severe mpox. The systematic review and meta-analysis by Pischel et al.^5^, provides a synthesis of the available data on MVA-BN protection against progression to severe outcomes among those who have breakthrough infections after vaccination. We can therefore combine our estimates of vaccine effectiveness against acquisition of infection with those from Pischel et al.^5^ to estimate overall vaccine effectiveness against severe disease. That is, if protection from acquisition is given by our estimates of vaccine effectiveness $V{E}_{1}\left(t\right)$ and $V{E}_{2,s}(t)$, after 1 and 2 doses (separated by $s$ days), respectively, and if protection against progression to severe outcomes (given breakthrough infection) is given by $V{E}_{p}$ from Pischel et al.^5^ (where ${V}{E}_{p}=66 \%$), and if we assume this protection against progression to severe outcomes is independent of (i) the number of doses an individual has had; (ii) antibody level; (iii) time since vaccination; and (iv) spacing between doses, we can calculate the protection against acquisition of severe mpox after 1 and 2 dose vaccination as,

24 $$\begin{array}{c}V{E}_{1}^{{severe}}\left(t\right)=1-\left(1-V{E}_{1}\left(t\right)\right)\left(1-V{E}_{p}\right)\,\end{array}$$

and

25 $$\begin{array}{c}V{E}_{2,s}^{{severe}}\left(t\right)=1-\left(1-V{E}_{2,s}\left(t\right)\right)\left(1-V{E}_{p}\right).\,\end{array}$$

Therefore, in order to calculate the ratio of severe cases averted, we recompute the equations and bounds on the ratio of cases averted, using these functions of vaccine effectiveness against severe mpox in place of our previous functions against acquisition of mpox. That is, we replace $V{E}_{1}\left(t\right)$ by $V{E}_{1}^{{severe}}\left(t\right)$ and $V{E}_{2,s}\left(t\right)$ by $V{E}_{2,s}^{{severe}}\left(t\right)$ from Eqs. 24 and 25 above. Note that we do not take into account uncertainty in the estimate for the $V{E}_{p}$ in this analysis.

### Computing the ratio of cases averted, bounds on ratio of cases averted and critical risk ratios using posterior sampling of vaccine effectiveness

In order to obtain estimates for the ratio of cases averted, critical risk thresholds and the bounds on these quantities (in the case of a time-varying force of infection), we need estimates for the vaccine effectiveness and vaccine effectiveness waning over time. These were taken from the our previous study where models were fitted to data on vaccine effectiveness and antibody titres following vaccination using a Bayesian framework^4^ (the relevant information from this previous study is reported in the supplementary information). Specifically, we drew posterior samples of vaccine effectiveness over time (*n* = 20,000) from the previously fitted models using the default HMC sampler in the RStan package^39,40^. Applying our equations for the ratio of cases averted and critical risk threshold (Eqs. (7–10,17,18,21–23)), we obtain posterior samples for these quantities. These samples are then summarised by the median for our central estimate and 2.5th and 97.5th percentiles for the 95% credible intervals. Note that when computing 95% credible intervals for the upper and lower bounds on the ${RC}{A}_{m}(t)$ from Eqs. (21) and (22) (and when calculating bounds on the risk threshold from these equations, as described in “Calculating the risk threshold”**)**, the 2.5th percentile of the lower bound (Eq. (22)) was used as the bottom of the 95% credible interval and the 97.5th percentile of the upper bound (Eq. (21)) was used as the upper 95% credible interval (Figs. 2, 4 and 5c).

## Supplementary information

Supplementary Information

## Data Availability

No primary data was used in the modelling or analysis presented in this work, but all relevant data or model outputs from previous studies, which are used in this study, are made available in the GitHub repository https://github.com/iap-sydney/Mpox_optimal_dosing.git.

## Code availability

All code are made available in the GitHub repository https://github.com/iap-sydney/Mpox_optimal_dosing.git.
