# Supplementary material for: Optimal deployment of limited vaccine supplies to control mpox outbreaks
Source: NPJ Vaccines. 2025 Nov 20;10:240. doi: 10.1038/s41541-025-01289-5 (PMC12635158; doi:10.1038/s41541-025-01289-5)
Supplement: Supplementary file 1 — Supplementary Information [file 41541_2025_1289_MOESM1_ESM.pdf]

Supplementary Information

Expanded description of the model derivation for the ratio of cases averted

Here we derive the ratio for cases averted for the scenario with variable force of infection (equation (17) in main text). We start with the model equations (equations (11) and (12) in the manuscript),

$$\frac{dS_u}{dt} = -r(t)S_u, \tag{1}$$

$$\frac{dS_v}{dt} = -\big(1 - VE(t)\big)r(t)S_v, \tag{2}$$

and the cumulative number of cases (equations (15) and (16) in the manuscript),

$$I_u(t) = S_v(0)\left(1 - e^{-\int_0^t r(\lambda)\,d\lambda}\right), \tag{3}$$

$$I_v(t) = S_v(0)\left(1 - e^{-\int_0^t r(\lambda)(1-VE(\lambda))\,d\lambda}\right), \tag{4}$$

we derive the ratio of cases averted. To derive this ratio, we consider three scenarios for vaccine deployment

i. Baseline reference scenario

First, as a reference, we consider a scenario of a naïve population who do not receive vaccination. Under this scenario a group of $2N$ naïve individuals are exposed to the force of infection $r(t)$. Using equation (3) above, and noting that in our case we have $S_u(0) = 2N$, the total number of cases occurring by time t is given by,

$$I_{base}(t) = 2N\left(1 - e^{-\int_0^t r(\lambda)d\lambda}\right) \tag{5}$$

ii. Two-dose scenario

Under this scenario a group of N individuals who received a first vaccine dose s days earlier are given a second dose, and as a result a group of N naïve individuals are left unvaccinated. All individuals are exposed to a force of infection $r(t)$. The second dose is administered at the time of the decision point $t = 0$ and the vaccine effectiveness in this doubly vaccinated group is given by $VE_{2,s}(t)$, where s is the number of days between the first and second dose (since VE is influenced by dose spacing). Thus, from equation (3) and (4) above, and noting that in our case we have $S_v(0) = S_u(0) = N$, the total number of cases occurring by time t is given by

$$I_{2dose}(t) = N\left(2 - e^{-\int_0^t r(\lambda)d\lambda} - e^{-\int_0^t r(\lambda)\left(1-VE_{2,s}(\lambda)\right)d\lambda}\right). \tag{6}$$

The number of cases averted in the two-dose scenario can be calculated by comparing the total number of cases occurring under this scenario with the number of cases occurring under the baseline scenario. Therefore, the number of cases averted under the two-dose scenario, $CA_2(t)$, is given by:

$$CA_2(t) = I_{base}(t) - I_{2dose}(t) = N\left(e^{-\int_0^t r(\lambda)\left(1-VE_{2,s}(\lambda)\right)d\lambda} - e^{-\int_0^t r(\lambda)d\lambda}\right). \tag{7}$$

iii. One dose scenario

Next, we consider the scenario where the N doses are deployed as first doses to a group of naïve individuals. This group will have a vaccine efficacy at time t that is given by $VE_1(t)$. This leaves a group of N individuals who were given their first dose s days earlier, and who will have a vaccine efficacy at time t that is given by $VE_1(t+s)$ (since their protection will have waned in the s days since the vaccine was administered. From equation (4) it follows that the total number of cases occurring by time t is given by,

$$I_{1dose}(t) = N\left(2 - e^{-\int_0^t r(\lambda)(1-VE_1(\lambda))d\lambda} - e^{-\int_0^t r(\lambda)(1-VE_1(\lambda+s))d\lambda}\right). \tag{8}$$

The number of cases averted in the one dose scenario can be calculated by comparing the total number of cases under this scenario with the number of cases under the baseline scenario. Therefore, the number of cases averted under the one dose scenario, $CA_1(t)$, is given by:

$$\begin{aligned} CA_1(t) &= I_{base}(t) - I_{1dose}(t) \\ &= N\left(e^{-\int_0^t r(\lambda)(1-VE_1(\lambda))d\lambda} + e^{-\int_0^t r(\lambda)(1-VE_1(\lambda+s))d\lambda} \right. \\ &\quad \left. - 2e^{-\int_0^t r(\lambda)d\lambda}\right). \end{aligned} \tag{9}$$

We derive the ratio of cases averted between the one and two dose scenarios in this case of a homogenous population, $RCA_h(t)$, (equation (17) of the main text) by comparing equations (8) and (9) and arrive at the relationship

$$RCA_h(t) = \frac{CA_1(t)}{CA_2(t)} = \frac{\left(2 - e^{-\int_0^t r(\lambda)(1-VE_1(\lambda))d\lambda} - e^{-\int_0^t r(\lambda)(1-VE_1(\lambda+s))d\lambda}\right)}{\left(2 - e^{-\int_0^t r(\lambda)d\lambda} - e^{-\int_0^t r(\lambda)\left(1-VE_{2,s}(\lambda)\right)d\lambda}\right)}. \tag{10}$$

When this ratio is more than 1, the number of cases averted by the one dose strategy is more than the number of cases averted by the two-dose strategy, and when this ratio is less than one then the two-dose strategy averts more cases than the one dose strategy.

Expanded description of the model derivation for the ratio of cases averted in a mixed population of high and low-risk groups

The model used in the methods (equation (19) of main text) that describes the ratio of cases averted when considering two subpopulations with different risks and variable force of infection is derived below. This model assumes that for one reason or another vaccinating more high-risk individuals with a first dose is not possible (e.g. all those willing to be vaccinated in this category have received a first dose). Therefore, this model captures the decision of whether to give those high-risk individuals who have had one dose (at least 28 days earlier) a second dose or whether to deploy first doses into the lower risk population.

We assume high risk individuals have an F fold higher risk of infection, i.e. $r_h(\lambda) = Fr_l(\lambda)$, for all $\lambda \in [0, t]$ where $r_h(\lambda)$ is the force of infection in the high risk group and $F \geq 1$ (i.e. there is a constant ratio in the force of infection between high and low-risk individuals over

time). In this case, the one dose strategy involves leaving high risk individuals with the waning protection of their first dose, administered s days earlier, and vaccinating lower risk individuals with a first dose. The two-dose strategy involves administering doses as second doses to the high-risk individuals and leaving lower risk individuals unvaccinated. The ratio of cases averted under these two scenarios with mixed risk populations is only slightly different to that of equation (10) (equation (17) in main text), and is given by,

$$RCA_m(t) = \frac{e^{-\int_0^t r_l(\lambda)d\lambda} + e^{-F\int_0^t r_l(\lambda)d\lambda} - e^{-\int_0^t r_l(\lambda)(1-VE_1(\lambda))d\lambda} - e^{-F\int_0^t r_l(\lambda)(1-VE_1(\lambda+s))d\lambda}}{e^{-F\int_0^t r_l(\lambda)d\lambda} - e^{-F\int_0^t r_l(\lambda)\left(1-VE_{2,s}(\lambda)\right)d\lambda}}. \quad (11)$$

where $r_l(\lambda) \geq 0$ and is the force of infection (without vaccination) at time λ, t is the follow-up time of our comparison of strategies, F is the fold-higher risk in a high risk subgroup compared with a lower risk subgroup, $VE_1(\lambda)$ is the vaccine effectiveness of 1 dose at time t after vaccination, $VE_{2,s}(\lambda)$ is the vaccine effectiveness of 2 doses at time t after the second dose, $s > 0$ is a time interval between the first deployment of vaccines and the second deployment of vaccines. We further constrain $r_l(\lambda)$, such that $\int_0^t r_l(\lambda)d\lambda > 0$ (i.e. the force of infection over the entire time period cannot be equal to zero), so cases over the total time period (without vaccination) cannot be zero. Of note, $RCA_h(t) = RCA_m(t)$ when $F = 1$.

We now re-parameterise equation (11). Let

$$\overline{r_t} = \int_0^t r_l(\lambda)d\lambda \quad (12)$$

and

$$h(\lambda) = \frac{r_l(\lambda)}{\overline{r_t}}. \quad (13)$$

We note that $h(\lambda)$ represents the force of infection at time $\lambda \in [0, t]$ normalised by the cumulative incidence over the entire interval $[0, t]$ and has the property

$$\int_0^t h(\lambda)d\lambda = \frac{1}{\overline{r_t}}\int_0^t r_l(\lambda)d\lambda = 1. \quad (14)$$

Now, notice equation (11) can be written as,

$$RCA_m(t) = \frac{e^{-\overline{r_t}} + e^{-F\overline{r_t}} - e^{-\overline{r_t}\int_0^t h(\lambda)(1-VE_1(\lambda))d\lambda} - e^{-F\overline{r_t}\int_0^t h(\lambda)(1-VE_1(\lambda+s))d\lambda}}{e^{-F\overline{r_t}} - e^{-F\overline{r_t}\int_0^t h(\lambda)\left(1-VE_{2,s}(\lambda)\right)d\lambda}} \quad (15)$$

Further, let $a_t = e^{-F\overline{r_t}}$, which represents the proportion of high-risk susceptible individuals in an unvaccinated population who would remain uninfected by time t (given the force of infection). With this re-parameterisation we see,

$$RCA_m(t) = \frac{a_t^{\frac{1}{F}} + a_t - a_t^{\left(\frac{1}{F}\right)\int_0^t h(\lambda)(1-VE_1(\lambda))d\lambda} - a_t^{\int_0^t h(\lambda)(1-VE_1(\lambda+s))d\lambda}}{a_t - a_t^{\int_0^t h(\lambda)\left(1-VE_{2,s}(\lambda)\right)d\lambda}}. \quad (16)$$

where $\int_0^t h(\lambda)d\lambda = 1, 0 < a_t \leq 1$. Rearranging we obtain

$$RCA_m(t) = \frac{a_t^{\frac{1}{F}} + a_t - a_t^{\left(\frac{1}{F}\right)} a_t^{-\left(\frac{1}{F}\right)\int_0^t h(\lambda)(VE_1(\lambda))d\lambda} - a_t\, a_t^{-\int_0^t h(\lambda)(VE_1(\lambda+s))d\lambda}}{a_t - a_t\, a_t^{-\int_0^t h(\lambda)\left(VE_{2,s}(\lambda)\right)d\lambda}}. \quad (17)$$

Finding bounds on the ratio of cases averted when the force of infection and vaccine effectiveness are time varying

In order to consider the ratio of cases averted under the two strategies for vaccine effectiveness that wanes over time we note that while the exact ratio of cases averted will depend on the choice of the time-varying force of infection $h(\lambda)$, it is possible to determine upper and lower bounds on $RCA_m(t)$ that are independent of the exact function $h(\lambda)$. We derive these bounds below (equations (21) and (22) from the methods of the main text). Considering the numerator and denominator of equation (17) we define $f(p)$ and $g(p)$ as

$$f(p) = -a_t^{\left(\frac{1}{F}\right)} a_t^{-p\left(\frac{1}{F}\right)\int_0^t h(\lambda)(VE_1(\lambda))d\lambda} - a_t\, a_t^{-p\int_0^t h(\lambda)(VE_1(\lambda+s))d\lambda} \quad (18)$$

and

$$g(p) = -a_t\, a_t^{-p\int_0^t h(\lambda)\left(VE_{2,s}(\lambda)\right)d\lambda} \quad (19)$$

for $p \in [0,1]$.

Notice that,

$$f(1) - f(0) = a_t^{\frac{1}{F}} + a_t - a_t^{\frac{1}{F}} a_t^{-\left(\frac{1}{F}\right)\int_0^t h(\lambda)(VE_1(\lambda))d\lambda} - a_t\, a_t^{-\int_0^t h(\lambda)(VE_1(\lambda+s))d\lambda} \quad (20)$$

and

$$g(1) - g(0) = a_t - a_t\, a_t^{-\int_0^t h(\lambda)\left(VE_{2,s}(\lambda)\right)d\lambda}. \quad (21)$$

Therefore,

$$RCA_m(t) = \frac{f(1) - f(0)}{g(1) - g(0)}. \quad (22)$$

Since, $f(p)$ and $g(p)$ are continuous and differentiable on the interval on the interval $[0,1]$, using Cauchy's Mean Value theorem[2] it follows that there exists a $c \in [0,1]$ such that,

$$\frac{f'(c)}{g'(c)} = \frac{f(1) - f(0)}{g(1) - g(0)}. \quad (23)$$

Thus, there is a $c \in [0,1]$ such that,

$$RCA_m(t) = \frac{f'(c)}{g'(c)}. \quad (24)$$

We note that the derivatives of $f(p)$ and $g(p)$ with respect to p are

$$f'(p) = \log(a_t)\, a_t^{\left(\frac{1}{F}\right)} \left(\frac{1}{F}\right) \int_0^t h(\lambda)\big(VE_1(\lambda)\big) d\lambda \;\; a_t^{-p\left(\frac{1}{F}\right)\int_0^t h(\lambda)(VE_1(\lambda))d\lambda}$$

$$+ \log(a_t)\, a_t \int_0^t h(\lambda)\big(VE_1(\lambda+s)\big) d\lambda \;\; a_t^{-p\int_0^t h(\lambda)(VE_1(\lambda+s))d\lambda} \tag{25}$$

and

$$g'(p) = \log(a_t)\, a_t \int_0^t h(\lambda)\left(VE_{2,s}(\lambda)\right) d\lambda \;\; a_t^{-p\int_0^t h(\lambda)\left(VE_{2,s}(\lambda)\right)d\lambda}, \tag{26}$$

respectively. Therefore, there is a $c \in [0,1]$, such that,

$$RCA_m(t) = \frac{\left(\frac{a^{\left(\frac{1}{F}\right)-1}}{F}\right)\int_0^t h(\lambda)\big(VE_1(\lambda)\big)d\lambda \;\; a^{-c\left(\frac{1}{F}\right)\int_0^t h(\lambda)(VE_1(\lambda))d\lambda}}{\int_0^t h(\lambda)\left(VE_{2,s}(\lambda)\right)d\lambda \;\; a^{-c\int_0^t h(\lambda)\left(VE_{2,s}(\lambda)\right)d\lambda}}$$

$$+ \frac{\int_0^t h(\lambda)\big(VE_1(\lambda+s)\big)d\lambda \;\; a^{-c\int_0^t h(\lambda)(VE_1(\lambda+s))d\lambda}}{\int_0^t h(\lambda)\left(VE_{2,s}(\lambda)\right)d\lambda \;\; a^{-c\int_0^t h(\lambda)\left(VE_{2,s}(\lambda)\right)d\lambda}} \tag{27}$$

Simplifying equation (27) we obtain

$$RCA_m(t) = \left(\frac{a_t^{\left(\frac{1}{F}\right)-1}}{F}\right) \frac{\int_0^t h(\lambda)\big(VE_1(\lambda)\big)d\lambda}{\int_0^t h(\lambda)\left(VE_{2,s}(\lambda)\right)d\lambda}\, a_t^{c\int_0^t h(\lambda)\left(VE_{2,s}(\lambda)-\frac{1}{F}VE_1(\lambda)\right)d\lambda}$$

$$+ \frac{\int_0^t h(\lambda)\big(VE_1(\lambda+s)\big)d\lambda}{\int_0^t h(\lambda)\left(VE_{2,s}(\lambda)\right)d\lambda}\, a_t^{c\int_0^t h(\lambda)\left(VE_{2,s}(\lambda)-VE_1(\lambda+s)\right)d\lambda}. \tag{28}$$

Noting that,

$$a_t \leq 1$$

$$\int_0^t h(\lambda)\left(VE_{2,s}(\lambda) - \frac{1}{F}VE_1(\lambda)\right)d\lambda \geq 0 \tag{29}$$

and

$$\int_0^t h(\lambda)\left(VE_{2,s}(\lambda) - VE_1(\lambda+s)\right)d\lambda \geq 0 \tag{30}$$

it follows that

$$a_t^{c\int_0^t h(\lambda)\left(VE_{2,s}(\lambda)-\frac{1}{F}VE_1(\lambda)\right)d\lambda} \leq a_t^0 = 1 \tag{31}$$

and

$$a_t^{c\int_0^t h(\lambda)\left(VE_{2,s}(\lambda)-\frac{1}{F}VE_1(\lambda)\right)d\lambda} \geq a_t^{\int_0^t h(\lambda)\left(VE_{2,s}(\lambda)-\frac{1}{F}VE_1(\lambda)\right)d\lambda} \tag{32}$$

since $c \in [0,1]$.

Thus,

$$RCA_m(t) \leq \left(\frac{a_t^{\left(\frac{1}{F}\right)-1}}{F}\right)\frac{\int_0^t h(\lambda)\big(VE_1(\lambda)\big)d\lambda}{\int_0^t h(\lambda)\left(VE_{2,s}(\lambda)\right)d\lambda} + \frac{\int_0^t h(\lambda)\big(VE_1(\lambda+s)\big)d\lambda}{\int_0^t h(\lambda)\left(VE_{2,s}(\lambda)\right)d\lambda} \tag{33}$$

and

$$\begin{aligned} RCA_m(t) \geq & \left(\frac{a_t^{\left(\frac{1}{F}\right)-1}}{F}\right)\frac{\int_0^t h(\lambda)\big(VE_1(\lambda)\big)d\lambda}{\int_0^t h(\lambda)\left(VE_{2,s}(\lambda)\right)d\lambda} a_t^{\int_0^t h(\lambda)\left(VE_{2,s}(\lambda)-\frac{1}{F}VE_1(\lambda)\right)d\lambda} \\ & + \frac{\int_0^t h(\lambda)\big(VE_1(\lambda+s)\big)d\lambda}{\int_0^t h(\lambda)\left(VE_{2,s}(\lambda)\right)d\lambda} a_t^{\int_0^t h(\lambda)\left(VE_{2,s}(\lambda)-VE_1(\lambda+s)\right)d\lambda}. \end{aligned} \tag{34}$$

Again, applying Cauchy's mean value theorem, it follows that for two functions,

$$\gamma(\rho) = \left(\frac{a_t^{\left(\frac{1}{F}\right)-1}}{F}\right)\int_0^\rho h(\lambda)\big(VE_1(\lambda)\big)d\lambda + \int_0^\rho h(\lambda)\big(VE_1(\lambda+s)\big)d\lambda \tag{35}$$

and

$$\phi(\rho) = \int_0^\rho h(\lambda)\left(VE_{2,s}(\lambda)\right)d\lambda \tag{36}$$

there exists a $\mu \in [0,t]$, such that

$$\begin{aligned} RCA_m(t) & \leq \left(\frac{a_t^{\left(\frac{1}{F}\right)-1}}{F}\right)\frac{\int_0^t h(\lambda)\big(VE_1(\lambda)\big)d\lambda}{\int_0^t h(\lambda)\left(VE_{2,s}(\lambda)\right)d\lambda} + \frac{\int_0^t h(\lambda)\big(VE_1(\lambda+s)\big)d\lambda}{\int_0^t h(\lambda)\left(VE_{2,s}(\lambda)\right)d\lambda} \\ & = \frac{\gamma'(\mu)}{\phi'(\mu)} \\ & = \frac{\left(\frac{a_t^{\left(\frac{1}{F}\right)-1}}{F}\right)VE_1(\mu) + VE_1(\mu+s)}{VE_{2,s}(\mu)}. \end{aligned} \tag{37}$$

Therefore,

$$RCA_m(t) \leq \max_{\mu\in[0,t]} \frac{\left(\frac{a_t^{\left(\frac{1}{F}\right)-1}}{F}\right)VE_1(\mu) + VE_1(\mu+s)}{VE_{2,s}(\mu)}. \tag{38}$$

Similarly, for two functions

$$\nu(\rho) = \left(\frac{a_t^{\left(\frac{1}{F}\right)-1}}{F}\right)\int_0^{\rho} h(\lambda)\big(VE_1(\lambda)\big)d\lambda\, a_t^{\int_0^t h(\lambda)\left(VE_{2,s}(\lambda)-\frac{1}{F}VE_1(\lambda)\right)d\lambda}$$

$$+\int_0^{\rho} h(\lambda)\big(VE_1(\lambda+s)\big)d\lambda\, a_t^{\int_0^t h(\lambda)\left(VE_{2,s}(\lambda)-VE_1(\lambda+s)\right)d\lambda} \qquad (39)$$

and

$$\omega(\rho) = \int_0^{\rho} h(\lambda)\left(VE_{2,s}(\lambda+s)\right)d\lambda \qquad (40)$$

there exists a $\theta \in [0,t]$ such that

$$RCA_m(t) \geq \left(\frac{a_t^{\left(\frac{1}{F}\right)-1}}{F}\right)\frac{\int_0^t h(\lambda)\big(VE_1(\lambda)\big)d\lambda}{\int_0^t h(\lambda)\left(VE_{2,s}(\lambda)\right)d\lambda}\, a_t^{\int_0^t h(\lambda)\left(VE_{2,s}(\lambda)-\frac{1}{F}VE_1(\lambda)\right)d\lambda} +$$

$$\frac{\int_0^t h(\lambda)\big(VE_1(\lambda+s)\big)d\lambda}{\int_0^t h(\lambda)\left(VE_{2,s}(\lambda)\right)d\lambda}\, a_t^{\int_0^t h(\lambda)\left(VE_{2,s}(\lambda)-VE_1(\lambda+s)\right)d\lambda}$$

$$= \frac{\nu'(\theta)}{\omega'(\theta)}$$

$$= \left(\frac{a_t^{\left(\frac{1}{F}\right)-1}}{F}\right)\frac{VE_1(\theta)}{VE_{2,s}(\theta)}\, a_t^{\int_0^t h(\lambda)\left(VE_{2,s}(\lambda)-\frac{1}{F}VE_1(\lambda)\right)d\lambda} + \frac{VE_1(\theta+s)}{VE_{2,s}(\theta)}\, a_t^{\int_0^t h(\lambda)\left(VE_{2,s}(\lambda)-VE_1(\lambda+s)\right)d\lambda}. \qquad (41)$$

Also, noting that

$$a_t^{\max_{\lambda\in[0,t]}\left(VE_{2,s}(\lambda)-\frac{1}{F}VE_1(\lambda)\right)} \leq a_t^{\int_0^t h(\lambda)\left(VE_{2,s}(\lambda)-\frac{1}{F}VE_1(\lambda)\right)d\lambda} \leq a_t^{\min_{\lambda\in[0,t]}\left(VE_{2,s}(\lambda)-\frac{1}{F}VE_1(\lambda)\right)} \qquad (42)$$

and

$$a_t^{\max_{\lambda\in[0,t]}\left(VE_{2,s}(\lambda)-VE_1(\lambda+s)\right)} \leq a_t^{\int_0^t h(\lambda)\left(VE_{2,s}(\lambda)-VE_1(\lambda+s)\right)d\lambda} \leq a_t^{\min_{\lambda\in[0,t]}\left(VE_{2,s}(\lambda)-VE_1(\lambda+s)\right)}, \qquad (43)$$

it follows that

$$RCA_m(t) \geq \min_{\theta\in[0,t]}\left(\left(\frac{a_t^{\left(\frac{1}{F}\right)-1}}{F}\right)\frac{VE_1(\theta)}{VE_{2,s}(\theta)}\, a_t^{\max_{\lambda\in[0,t]}\left(VE_{2,s}(\lambda)-\frac{1}{F}VE_1(\lambda)\right)}\right.$$

$$\left.+\frac{VE_1(\theta+s)}{VE_{2,s}(\theta)}\, a_t^{\max_{\lambda\in[0,t]}\left(VE_{2,s}(\lambda)-VE_1(\lambda+s)\right)}\right). \qquad (44)$$

Thus, to summarise from equation (38) we obtain an upper bound for $RCA_m(t)$, defined by

$$RCA_m(t) \leq \max_{\mu \in [0,t]} \frac{\left(\frac{a_t^{\left(\frac{1}{F}\right)-1}}{F}\right) VE_1(\mu) + VE_1(\mu + s)}{VE_{2,s}(\mu)}, \tag{45}$$

and from equation (44) we derive a lower bound for $RCA_m(t)$ defined by

$$RCA_m(t) \geq \min_{\theta \in [0,t]} \left(\left(\frac{a_t^{\left(\frac{1}{F}\right)-1}}{F}\right) \frac{VE_1(\theta)}{VE_{2,s}(\theta)} a_t^{\max_{\lambda \in [0,t]}\left(VE_{2,s}(\lambda) - \frac{1}{F} VE_1(\lambda)\right)} + \frac{VE_1(\theta + s)}{VE_{2,s}(\theta)} a_t^{\max_{\lambda \in [0,t]}\left(VE_{2,s}(\lambda) - VE_1(\lambda + s)\right)} \right). \tag{46}$$

Refining the upper and lower bounds on RCA

In some regions of the parameter space for F and a we noticed these bounds could become quite wide. Thus, we refined the bounds by noting that

$$VE_1(t) \leq VE_1(\lambda) \leq VE_1(0),$$

$$VE_1(t + s) \leq VE_1(\lambda + s) \leq VE_1(0 + s)$$

and

$$VE_2(t) \leq VE_2(\lambda) \leq VE_2(0)$$

for all $\lambda \in [0, t]$.

It also follows that,

$$RCA_m(t) = \frac{a_t^{\frac{1}{F}} + a_t - a_t^{\left(\frac{1}{F}\right)} a_t^{-\left(\frac{1}{F}\right)\int_0^t h(\lambda)(VE_1(\lambda))d\lambda} - a_t\, a_t^{-\int_0^t h(\lambda)(VE_1(\lambda + s))d\lambda}}{a_t - a_t\, a_t^{-\int_0^t h(\lambda)\left(VE_{2,s}(\lambda)\right)d\lambda}} \tag{47}$$

is bounded by

$$RCA_m(t) \leq \frac{a_t^{\frac{1}{F}} + a_t - a_t^{\left(\frac{1}{F}\right)} a_t^{-\left(\frac{1}{F}\right)VE_1(t)} - a_t\, a_t^{-VE_1(t+s)}}{a_t - a_t\, a_t^{-VE_{2,s}(0)}} \tag{48}$$

and

$$RCA_m(t) \geq \frac{a_t^{\frac{1}{F}} + a_t - a_t^{\left(\frac{1}{F}\right)} a_t^{-\left(\frac{1}{F}\right)VE_1(0)} - a_t\, a_t^{-VE_1(s)}}{a_t - a_t\, a_t^{-VE_{2,s}(t)}}. \tag{49}$$

Therefore, for any given $a_t \leq 1$ and $F \geq 1$

$$RCA_m(t) \leq \min\left(\frac{a_t^{\frac{1}{F}} + a_t - a_t^{\left(\frac{1}{F}\right)} a_t^{-\left(\frac{1}{F}\right)VE_1(t)} - a_t\, a_t^{-VE_1(t+s)}}{a_t - a_t\, a_t^{-VE_{2,s}(0)}}, \max_{\mu\in[0,t]} \frac{\left(\frac{a_t^{\left(\frac{1}{F}\right)-1}}{F}\right) VE_1(\mu) + VE_1(\mu+s)}{VE_{2,s}(\mu)} \right) \quad (50)$$

and

$$RCA_m(t) \geq \max\left(\frac{a_t^{\frac{1}{F}} + a_t - a_t^{\left(\frac{1}{F}\right)} a_t^{-\left(\frac{1}{F}\right)VE_1(0)} - a_t\, a_t^{-VE_1(s)}}{a - a\, a^{-VE_{2,s}(t)}}, \min_{\theta\in[0,t]} \left(\left(\frac{a_t^{\left(\frac{1}{F}\right)-1}}{F}\right) \frac{VE_1(\theta)}{VE_{2,s}(\theta)} a_t^{\max_{\lambda\in[0,t]}\left(VE_{2,s}(\lambda) - \frac{1}{F}VE_1(\lambda)\right)} + \frac{VE_1(\theta+s)}{VE_{2,s}(\theta)} a_t^{\max_{\lambda\in[0,t]}\left(VE_{2,s}(\lambda) - VE_1(\lambda+s)\right)} \right) \right). \quad (51)$$

These inequalities provide bounds on the ratio of cases averted that depend only on a_t, F, s and the functions describing vaccine effectiveness over time. The bounds for the ratio of cases averted in the homogenous case, $RCA_h(t)$, can be obtained by setting $F = 1$ in equations (50) and (51) (Equations (21) and (22) in the main text).

Description of the previous model used to estimate vaccine effectiveness and predict VE waning over time from Berry et al.

Previously, we conducted a systematic review and meta-analysis of vaccine effectiveness (VE) and immunogenicity after vaccination[1]. In this meta-analysis we used a double exponential decay (Equation (52)) to model the waning of antibody titres.

The antibody titre at some time t in an individual i after the peak response after the vaccine regimen w is modelled by

$$b_w^i(t) = b_{0,w}^i\left(f_w e^{-\delta_1 t} + (1 - f_w)e^{-\delta_2 t}\right), \quad (52)$$

where $b_{0,w}^i$ is the initial antibody titre for individual i and f_w is the proportion of fast decaying antibodies for the vaccine regimen w, and δ_1, δ_2 are the decay rates with $\delta_1 > \delta_2$. We assume that the initial antibody titers $b_{0,w}^i$, follow a log-normal distribution across the population, such that, $log_{10}\left(b_{0,w}^i\right) \sim N(\mu_w, \sigma_w)$. Each individual's antibody titre decays following equation (49).

In addition to the waning of antibody titres, a logistic relation was used to relate binding antibody titres to the VE. Firstly, individual protection, $p(x)$, is assumed to be dependent on the logarithm of the antibody titre, x, following the logistic relationship,

$$p(x) = \frac{1}{1 + e^{-kx+A}}, \tag{53}$$

where, k represents the steepness of the logistic function and $\frac{A}{k}$ is the antibody titre associated with 50% protection. Therefore, for an individual, their protection at time, t, is given by, $p\left(log_{10}\, b_w^i\,(t)\right)$. As a result, the overall protection for a group of individuals is the average of $p\left(log_{10}\, b_w^i\,(t)\right)$ across the distribution of antibody titres in the population. To obtain the vaccine effectiveness for a vaccine regimen, w, we can average the protection across the population, which is given by, $VE_w(t) = E\left(p\left(log\left(b_w^i(t)\right)\right)\right)$, which is the expectation of the protection function across the distribution in initial antibody titres.

The parameters describing protection as a function of antibody titre (equation 53), and the antibody titre over time (equation (52)) were estimated in our previous meta-analysis by fitting these models to data on vaccine effectiveness and antibody titres using a Bayesian inference approach (for the full details including likelihoods and priors see the original meta-analysis[1]). The estimated parameters and credible intervals from this previous analysis[1] are provided in Table S1. Note that the only differences between the vaccine effectiveness across the different regimens, w, are the mean and standard deviation of the peak level of antibodies and the proportion of long- and short-lived antibodies (Table S1). Note that when we evaluate the vaccine effectiveness of two doses with a dose spacing of 26 weeks or 52 weeks, we assume the peak antibody titre and decay rates will be the same as that observed with a 104 week does spacing (Table S1). These estimates of vaccine effectiveness over time and for different regimens are used to estimate the $RCA_h(t)$ and $RCA_m(t)$, bounds on the ratio of cases averted (equations (50) and (51)) and risk thresholds.

Parameter		Symbol	Estimate (95% Credible Interval)
Logistic Slope		k	0.49 (0.21-0.79)
Logistic Constant		A	1.44 (0.89-1.92)
Initial log(GMT) mean	1-Dose	μ_w	1.90 (1.80-2.00)
	2-Dose (4 weeks)		2.83 (2.73-2.93)
	2-Dose (104 weeks)		3.32 (3.20-3.46)
Initial GMT standard deviation	1-Dose	σ_w	0.76 (0.74-0.78)
	2-Dose (4 weeks)		0.51 (0.50-0.52)
	2-Dose (104 weeks)		0.48 (0.44-0.52)
Slow-decay rate (long-lived antibodies)		δ_l	0.0028 (0.0007- 0.0050)
Fast-decay rate (short-lived antibodies)		δ_s	0.23 (0.20-0.27)
Proportion of fast decaying antibodies	1-Dose	f_w	0.89 (0.87-0.92)
	2-Dose (4 weeks)		0.94 (0.93-0.95)
	2-Dose (104 weeks)		0.74 (0.65-0.81)

Table S1: Median estimates of the model parameters using the posterior samples generated from our prior work[1].

References

1. Berry, M.T., *et al.* Predicting vaccine effectiveness for mpox. *Nat Commun* **15**, 3856 (2024).
2. Cates, D.M. *Cauchy's Calcul Infinitésimal*, (Springer, 2019).
